# Changes in well-being after myocardial infarction: Does coping matter?

**DOI:** 10.1007/s11136-016-1286-6

**Published:** 2016-04-05

**Authors:** Aleksandra Kroemeke

**Affiliations:** Department of Psychology, SWPS University of Social Sciences and Humanities, Chodakowska Street 19/31, 03-815 Warsaw, Poland

**Keywords:** Affect, Coping strategies, Mediation analysis, Male, Longitudinal studies

## Abstract

**Purpose:**

This study explores changes in emotional component of subjective well-being (SWB) of patients after their first myocardial infarction (MI) and two kinds of mechanisms: attribution of positive (PA) and negative (NA) affect and a mediation effect of coping.

**Methods:**

Affect and coping strategies (problem-, emotion-, and avoidance-focused) were assessed in 121 male patients (age 52.26 ± 7.08 years) a few days after the first MI and then 1 and 6 months later. The indicator of emotional SWB was affect balance, calculated as the ratio of PA to NA. Mediation was tested using the PROCESS macro.

**Results:**

The affect balance changed over time, from a predominance of negativity a few days post-MI to more positive 1 and 6 months later (*F*_2, 119_ = 21.87, *p* < 0.001). The results of parallel multiple mediation showed a mediating effect of emotion-focused coping on the changes in affect balance over time. Separate analyses for PA and NA showed the same results for NA. Problem-focused coping mediated PA changes in the early post-hospitalization period.

**Conclusions:**

Myocardial infarction may activate PA alongside the NA, but the predominance of PA over NA in both early and late post-hospitalization period was minimal. Affect balance appeared to be largely dependent on NA and its changes. Coping strategies partly mediated the changes in well-being, providing a basis for practical interventions.

## Introduction

The present study examines the role of coping strategies for stress that may account for changes in the emotional well-being of male myocardial infarction (MI) survivors in a 6-month follow-up period.

Experiencing a life-threatening illness such as MI is a highly stressful event that may have serious consequences for a patient’s well-being for a substantial period of time. For example, previous studies show that MI survivors usually experience negative affect (NA) [1], which coincides with limited physical functioning, cardiac complications [2, 3], and deterioration of quality of life [4, 5]. Although recent studies have found that highly stressful events can also be accompanied by positive states [6], there is little research on positive affect (PA) among MI survivors. The present study attempted to address this gap. The findings of other studies confirm the adaptive value of PA in various stressful situations [7, 8] including life-threatening illnesses such as HIV/AIDS [9], cancer [10], or heart failure [11]. Furthermore, studies have proven the independence of PA and NA, e.g., [12], which implies that both could be co-activated within one event. The utility of PA and NA in the coping with stress is demonstrated in the broaden-and-build theory of positive affect [13]. According to the theory, NA narrows one’s thought-action repertoire as it evaluates conditions as either beneficial or disadvantageous, and then results in a fight-or-flight reaction [13]. In contrast, PA signals the individual’s opening to new opportunities and broadens the scope of the available knowledge and activities, thus building up one’s personal resources, all of which broaden one’s thought-action repertoire [13]. Thus, the role of PA in coping efficacy is probably realized as an impact on the course of cognitive processes and as a stimulation of the individual’s activity in directly assisting the resolution of a difficult situation. All this may imply that the associations between affect and coping efforts (defined by Lazarus and Folkman [14]) are distinctive and that different kinds of coping regulate either one or both kinds of emotional states in stressful conditions. Coping–emotions relationship has been well studied. Overall, problem-focused coping (focused on modifying and managing the problem [14]) has been positively related to PA and negatively related to NA, whereas mood-regulating strategies (emotion- or avoidance-focused) have shown the opposite pattern in traditional cross-sectional and longitudinal studies on cancer [15] or everyday life [16, 17] as well as in intensive longitudinal research on headache [18], daily life [19], or meta-analyses (e.g., on coping with HIV [20]).

The search for such distinctive compounds has led to empirical focus on the impact of PA and NA separately. However, both valences of affect contribute to emotional components of subjective well-being (SWB) [21]. Moreover, for emotional SWB the predominance of PA over NA (i.e., affect balance or positivity) is more important. Therefore, the present study attempted to address this issue. The rationale for searching for such a proportion is (a) the independence of both affects and (b) their co-activation in the stress process. The added benefit of such an affect indicator is its usefulness for determining the effect of one affect above and beyond the second. In previous studies, affect balance has been positively related to problem-focused coping and negatively related to avoidance- or emotion-focused coping [22–24]. Prior studies did not concern affect balance changes in stressful events and its mechanisms.

The present investigation is maybe the first study aiming at examination of the dynamics of men’s affect balance and its mechanism within 6 months after MI. The first aim was to determine changes in affect balance and to test whether either one or both of the two emotional states—PA and NA—contribute to changes in positivity. The MI evokes an intense emotional response, which should decrease in time as patients’ physical condition changes—from MI treatment and closely monitored condition (hospitalization), via deprivation of a day and night care and simultaneously continuing weakness and other cardiac symptoms (early post-hospitalization period), to better health conditions and returning to work and other social roles (late post-hospitalization period). Therefore, an increase in affect balance, as well as in PA, and a decrease in NA from hospitalization via early to late post-hospitalization period were predicted. The second aim of the study was to test whether coping strategies (problem-, emotion-, and avoidance-focused) mediate changes in emotional SWB indicators after MI, as postulated by stress and coping theory [14]. It is suggested that coping is a key factor determining ones’ adjustment, which plays a mediating role in a stress process [14]. However, with respect to affect changes, previous findings have confirmed only the role of coping in PA changes [17]. Thus, it was hypothesized that problem-focused coping would mediate changes in affect balance and PA, whereas emotion- and avoidance-focused coping would mediate NA changes.

## Methods

### Participants

The sample comprised 121 male cardiac patients (age 52.26 ± 7.08 years) after their first MI. Participants were assessed three times: a few days after MI (T1; *N* = 141) and 1 month (T2; *N* = 126) and 6 months (T3; *N* = 121) later. Inclusion criteria were: first MI, absence of serious comorbidities (e.g., cancer, neurological, and mental disorders), and age ≤65 years. T1 was conducted in the one and the same cardiology clinic and T2 and T3 at the participants’ homes (response rate was 86 %; 11 participants refused to continue the study, 2 were hospitalized due to second MI or cancer in time of assessment; and contact was lost with 7). All the participants were treated with the percutaneous transluminal coronary angioplasty. Eighty-nine percent of the participants were in a stable relationship; 43 % had at least a secondary education, and 52 % of patients were employed before the MI. Nearly all the participants fell under Class I (50 % at T2 and 57 % at T3) or II (48 % at T2 and 35 % at T3) according to the New York Heart Association’s (NYHA) [25] functional classification system. Thus, for most of them, ordinary physical activity caused no (Class I) or slight (Class II) fatigue, palpitation, or dyspnea symptoms.

The study protocol was approved by the University’s Ethics Committee. Written informed consent was obtained from all the participants before data collection, and participation was voluntary. Paper-and-pencil questionnaires were filled out by patients independently. Data were collected during 1 year at the turn of 2006–2007. Sample attrition analyses indicated that the completers and non-completers did not differ in terms of socio-demographic, health-related, or any other major study’s variables.

### Measures

#### Affect balance and emotions

PA (Cronbach’s *α* from .90 at T1 to .91 at T3) and NA (*α* from .95 at T1 to .97 at T3) were assessed at all stages with the Positive and Negative Affect Schedule (PANAS) [26], adapted for use in Polish [27]. The two scales consist of 15 adjectives each (e.g., *Enthusiastic*, *Active*, *Nervous*, *Guilty*). Participants rated the extent to which they felt each of the 30 emotions right now (at the present moment), using a five-point Likert scale (total score: 15–75 for each of subscales). Affect balance (PA/NA) was computed by dividing the total PA by the total NA. Thus, a score of affect balance above “1” reflects predominance of PA over NA, while a score below “1” signals a predominance of NA over PA. Affect balance convergent validity was supported by significant negative correlations with depression scores (*r* values from −.22 to −.38).

#### Coping

Coping was assessed at all stages with Polish adaptation of the situation-specific Coping Inventory for Stressful Situations (CISS-S) [28, 29]. CISS-S measures problem-focused (seven items; e.g., *Focus on the problem and see how I can solve it*; *α* from .75 at T3 to .79 at T2), emotion-focused (seven items; e.g., *Become very upset*; *α* from .77 at T2 to .81 at T1 and 3), and avoidance-focused coping strategies (seven items; e.g., *Phone a friend*; *α* from .65 at T3 to .70 at T1). Participants rated the extent to which they undertook each behavior in a stressful situation (related to the MI and their somatic health) on a five-point scale (total score: 7–35 for each of the subscales).

### Statistical analysis

First, multivariate repeated measures analyses of variance (MANOVA) were conducted to test changes in affect balance and both affects (PA and NA) over the 6-month follow-up period. Then, parallel multiple mediation hypotheses were tested using a bootstrapping procedure, a nonparametric sampling method recommended for small and non-normally distributed samples [30]. The essence of the method is to generate a greater number of samples of the same size as the initial sample through random sampling with replacement (here *N* = 5000) and, for each sample created, to identify the range (using a bias-corrected and accelerated 95 % confidence interval; BCa 95 % CI) into which the value of a given parameter in a population is likely to fall [30]. If the BCa 95 % CI does not include zero, the indirect effect is significant and a mediation effect is demonstrated. The total indirect effect (the sum of indirect effects across all mediators in a certain model) and the specific indirect effect (the indirect effect of a particular mediator) were examined. The analysis was conducted with SPSS 22.0 with the PROCESS macro (model 4) [30]. Mean-centered variables were used. Overall, six models were tested: three analyzed changes in affect (PA/NA; PA; NA) between T1 and T2 and three compared T2 and T3. In the latter case, initial affective state was controlled for. Coping strategies mean values (T1–T2, T2–T3) were computed and included in the equation.

## Results

Descriptive statistics and correlations between affect indicators and coping are presented in Table 1. Affect balance scores above “1” were observed in 35.5 % (*n* = 43), 54.5 % (*n* = 66), and 57 % (*n* = 69) of participants, respectively, at T1, T2, and T3. In the remaining participants, this index was ≤1. The effect of socio-demographic variables on dependent variables was also tested; however, no significant relationships were found.

**Table 1:** Means, SD, and correlations of affect indicators and coping (*N* = 121)

	*M*	*SD*	2.	3.	4.	5.	6.	7.	8.	9.	10.	11.	12.	13.	14.	15.
1. PA/NA_1_	.94	.49	.32**	.33**	.46**	.22*	.12	−.68**	−.18	−.30**	−.05	−.14	−.30**	−.32**	−.01	.00
2. PA/NA_2_	1.20	.59		.50**	.10	.38**	.04	−.36**	−.74**	−.49**	.09	−.07	−.38**	−.45**	−.05	−.06
3. PA/NA_3_	1.41	.81			.00	.01	.42**	−.38**	−.47**	−.74**	−.17	−.20*	−.25**	−.40**	−.05	−.02
4. PA_1_	32.71	10.72				.56**	.24**	.21*	.28**	.13	.25**	.15	.18*	.12	.31**	.25**
5. PA_2_	36.94	9.47					.25**	.17	.19*	.15	.35**	.19*	.15	.07	.21*	.17
6. PA_3_	37.77	10.16						.14	.18*	.16	.11	.17	.23*	.23*	.21*	.19*
7. NA_1_	39.73	13.82							.53**	.51**	.19*	.23*	.52**	.51**	.25*	.16
8. NA_2_	35.81	13.74								.62**	.15	.17	.52**	.55**	.22*	.17
9. NA_3_	32.80	14.27									.28**	.34**	.44**	.59**	.19*	.14
10. PROB_1–2_	27.00	4.61										.84**	.28**	.25**	.32**	.28**
11. PROB_2–3_	26.14	4.86											.26**	.33**	.23*	.27**
12. EMOT_1–2_	16.17	5.35												.87**	.34**	.27**
13. EMOT_2–3_	15.30	5.47													.27**	.27**
14. AVOID_1–2_	13.99	4.09														.82**
15. AVOID_2–3_	13.27	3.99														

Index 1, 2, 3 = measurement occasion: T1, T2, T3, respectively

*PA/NA* Affect balance, *PA* positive affect, *NA* negative affect, *PROB* problem-focused coping, *EMOT* emotion-focused coping, *AVOID* avoidance-focused coping

* *p* < 0.05; ** *p* < 0.01

### Dynamics of positivity and affects after MI

To explore changes in affect balance, a MANOVA was conducted. The results showed a significant and linear increase in affect balance within 6 months post-MI (Wilks’s lambda *F*_2, 119_ = 21.87, *p* < 0.001, *η*^2^ = .27). In particular, the first stage was dominated by negativity (*t*_120_ = −4.21, *p* < 0.001); in the second stage, there were no significant differences between levels of PA and NA (*t*_120_ = .82, *p* = 0.412), while the third stage was dominated by PA (*t*_120_ = 3.39, *p* < 0.001). To recognize how affects changed over 6 months, MANOVAs for both PA and NA were also conducted. While NA systematically decreased over time after MI (Wilks’s lambda *F*_2, 119_ = 15.00, *p* < 0.001, *η*^2^ = .20), PA significantly increased only at T2 and remained at this level 5 months later (Wilks’s lambda *F*_2, 119_ = 14.53, *p* < 0.001, *η*^2^ = .19).

### Changes in positivity and affects: coping as a mediator

**Fig. 1:**
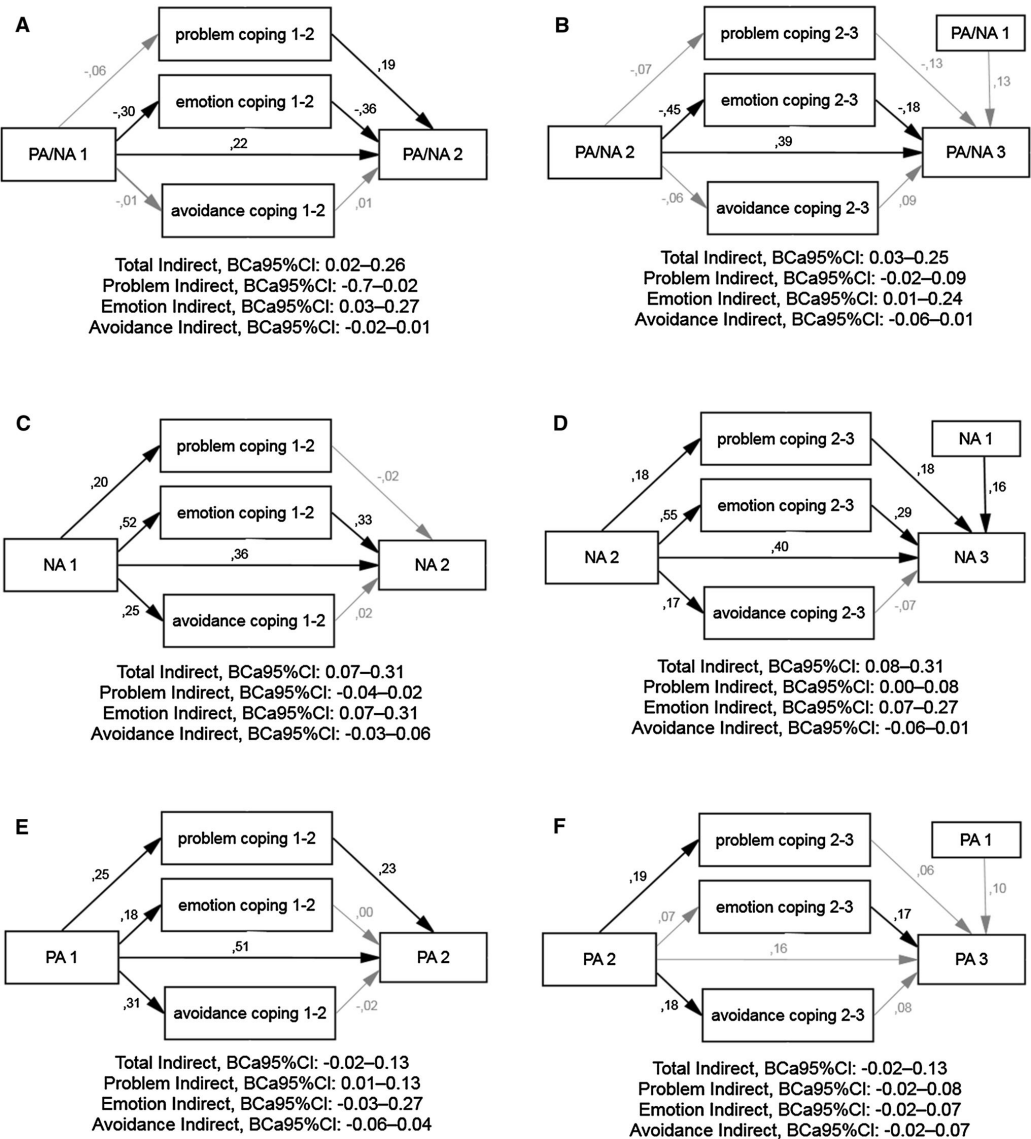
Panel of parallel multiple mediator models: associations between changes in affect balance (**a**, **b**), negative (**c**, **d**), and positive (**e**, **f**) affects via coping. Values presented are standardized coefficients, significant paths in *black*, significance defined as ≥.16 at *p* < 0.05; ≥.21 at *p* < 0.01; ≥.30 at *p* < 0.001. *BCa 95* *% CI* bias-corrected and accelerated 95 % confidence interval of mediation effect: total and specific via problem-, emotion-, and avoidance-focused coping

To determine the indirect effects of changes in PA/NA, PA, and NA over 6 months after MI via problem-, emotion- and avoidance-focused coping strategies, parallel multiple mediation analyses were conducted (Fig. 1).

The overall model for affect balance changes from T1 to T2 (PA/NA_1_, PA/NA_2_) was significant (*F*_4, 116_ = 8.55, *p* < 0.001) and predicted 23 % of the variance in PA/NA_2_ (Fig. 1a). PA/NA_1_ was directly (*B* = .27, SE = .10, *t* = 2.60, *p* = 0.010) and indirectly related to PA/NA_2_. The single specific indirect effect that was significant involved emotion-focused coping (*B* = .13, SE = .06, BCa 95 % CI .03–.27): Affect balance at T1 was negatively related to emotion-focused coping, which, in turn, was negatively related to PA/NA_2_. In addition, problem-focused coping significantly predicted PA/NA_2_.

Similar results were obtained for affect balance in the T2–T3 model (Fig. 1b). Analyzed variables explained 33 % of variance of PA/NA_3_ (*F*_5, 115_ = 11.26, *p* < 0.001). Apart from a positive association between both affect balances (*B* = .52, SE = .12, *t* = 4.32, *p* < 0.001), emotion-focused coping partially mediated these relationships (*B* = .11, SE = .06, BCa 95 % CI .01–.24).

For NA_1_ to NA_2_ changes, the overall model was significant (*F*_4, 116_ = 16.55, *p* < 0.001) and predicted 36 % of the variance in NA_2_ (Fig. 1c). NA_1_ significantly predicted all types of coping behaviors and NA_2_. In turn, only emotion-focused coping predicted negative affect; thus, partial mediation was found (direct effect: *B* = .36, SE = .09, *t* = 4.14, *p* < 0.001; specific indirect effect: *B* = .17, SE = .06, BCa 95 % CI .07–.31).

For NA_2_ to NA_3_ changes (Fig. 1d), the model explained 52 % of the variance in NA_3_ (*F*_5, 115_ = 24.66, *p* < 0.001). Besides a significant direct effect (*B* = .39, SE = .09, *t* = 4.57, *p* < 0.001), a specific indirect effect was found only for emotion-focused coping (*B* = .15, SE = .05, BCa 95 % CI .07–.27); however, problem-focused coping was also significantly related to NA_1_ and NA_2_. This result may be surprising, but as Hayes [31] has argued, significance or its lack for individual paths in a mediation model is irrelevant to the significance of indirect effects.

The association between positive states in the T1 to T2 period was direct (*B* = .45, SE = .07, *t* = 6.42, *p* < 0.001), but an indirect one was observed as well (Fig. 1e). This time, problem-focused coping was a significant mediator (*B* = .05, SE = .03, BCa 95 % CI .01–.13). Together, the variables predicted 36 % of the variance in PA_2_ (*F*_4, 116_ = 16.39, *p* < 0.001).

Despite a lack of changes in PA from T2 to T3, the PA_2_ to PA_3_ model was used to test for affect stability predictors. The direct effect of PA_2_ was not significant, *B* = .16, SE = .12, *t* = 1.45, *p* = 0.149. Only emotion-focused coping predicted PA_3_ (Fig. 1f). Moreover, positive relations were noted between PA_2_ and problem- and avoidance-focused coping (*F*_5, 115_ = 3.33, *p* = 0.007, adj. *R*^2^ = .13).

## Discussion and conclusions

With a focus on the positive facets of well-being, the aims of the study were to test changes in affect balance after the first MI, the dynamics of both PA and NA, and their contributions to affect balance changes. The mediation function of coping strategies in emotional state changes was also addressed. The findings revealed significant changes in men’s emotional well-being toward its improvements, which was related to short-term increase in PA and short- and long-term decrease in NA. Emotion- and problem-focused coping turned out to play a mediation role in this process.

The results showed that affect balance grew within 6 months after MI, but its dynamics over time was weak. It changed from a predominance of negativity a few days after MI (PA/NA = .94) to a more positive state a month later (PA/NA = 1.20) and continued in similar levels after 6 months (PA/NA = 1.41), but was still low. For comparison, mean affect balance (PA/NA just as in this study) among depressed patients undergoing therapy ranged from .76 (no remission) through 2.21 (typical remission) to 4.36 (optimal remission) [32]. In a study with patients with terminal cancer that included both PA and NA, the former prevails, but the ratio of PA to NA was comparable to those reported in the present study (range from 1.29 to 1.65 depending on the measurement occasion) [10].Therefore, although in this study affect balance was positive 1 and 6 months after MI, it was not large enough to distinguish the post-MI patients from clinically depressed individuals or terminally ill patients. Thus, it appears that it is not the mere dominance of one emotional state over another, but rather the size of the difference, that would allow to draw conclusions about the degree of adjustment to stressful events. There is, however, no agreement on the size of affect balance that would be considered most beneficial [33].

In regard to the mechanism of changes in post-MI affect balance, the results suggested that the increase in positivity was determined by a drop in NA, as well as an increase in PA 1 month after MI, and a subsequent further decline of NA in the 6-month follow-up. Namely, PA increased when the immediate threat to life disappeared and it remained at this level for the next 5 months. The level of NA was reduced throughout the 6-month period, and hence its role in affect balance seems dominant. However, it cannot be ruled out that the driving force in these changes was the earlier increase in PA. Indeed, it is believed that PA effects manifest themselves in the long term rather than short term [13]. The effect of an increase in PA on changes in NA requires further studies. Similar results were observed during exam experience [8]—negative emotions decreased over time, while positive emotions increased only after the test or the culmination of the transaction. In contrast, in a longitudinal study of cancer patients, neither PA nor NA changed significantly over time [10]. However, this study involved chronically ill patients whose somatic conditions did not change radically during the investigation.

In the present study, changes in post-MI affect balance were mediated by coping strategies, yet obtained results did not fully support all the hypotheses. Emotion-focused coping was found to mediate changes in affect balance. Separate analyses for PA and NA showed a similar pattern of results for NA, but its effect was opposite. That is, high affect balance decreased mood-regulating strategies, whose lower level in turn increased affect balance, regardless of a follow-up interval. In contrast, high NA increased emotion-focused coping, which further strengthened negative affect. Thus, emotion-focused coping appears to be a rather maladaptive strategy in both early (1 month) and late (6 months) post-MI-hospitalization periods. Additionally, it appears to be regulated by a negative state, but also indirectly influenced by positive affect (PA/NA).

The findings did not indicate the strategies responsible for strengthening affect balance, although the use of problem-focused strategies was partly connected with an increase in PA_2_. This result is similar to Yamasaki et al.’s findings [17]. However, it must be noted that all the indirect effects were only partial, which means some other mechanisms were also involved in changes in affect states. Taking into account individual paths of the multiple mediation models, obtained results were comparable with those obtained in other studies [17, 23, 24], which also noted positive associations between PA and emotion- and avoidance-focused coping [15, 16] or between NA and instrumental coping [19]. It is reasonable to assume that these findings result from the heterogeneity of problem-, emotion- and avoidance-focused strategies that represent a mixture of cognitive and behavioral coping strategies. Aforementioned varying processes (i.e., thinking and acting) in some cases may have contradictory effects on stress outcomes.

The limitations of the present study lie mainly in the characteristics of sample, its linear association assumptions, and its variable-centered perspective. The sample comprised mostly of poorly educated, middle-aged and retired men, which reflects the major characteristics of cardiac patients [34], but limits the generalizability of results. In addition, only linear associations between study variables were analyzed. Curvilinear associations could be present as well, particularly in light of the argument that at some point, more happiness might simple be “too much” [35]. Study of an optimal level of affect balance or mediation with nonlinear paths would require different methods (i.e., nonlinear models). Moreover, the study of affect may require different test procedures with day-by-day assessments (i.e., intensive longitudinal study). Finally, based on mean scores for the entire sample, according to a variable-centered approach [36], the study treated the sample as homogeneous with regard to its variables. The longitudinal patterns of change in affect and coping may be individualized (i.e., the sample may be heterogeneous in this regard), and such heterogeneity cannot be reflected by the mean scores. Of note, a 6-month follow-up period was analyzed, and it is suggested that future studies engage in longer follow-up assessment timeframes.

Despite these limitations, the present analyses make it clear that a life-threatening illness such as MI may in the same time generate both PA and NA. However, it must be recognized that even though the participants demonstrated improvement in emotional state in time, the predominance of PA over NA at 6 months post-MI was minimal. Furthermore, the affect balance over that time was largely dependent on the level of NA and its change. Coping strategies partly mediated the changes in emotional SWB—emotion-focused coping mediated affect balance and NA, whereas problem-focused coping indirectly affected PA. Furthermore, assessing affect balance may be a useful measure of emotional states or emotional component of well-being in a stress process. This issue, the present study notwithstanding, still requires exploration.

In the practical sense, it appears that interventions should be focused on the promotion and increase in affect balance that is, first and foremost, on enhancing PA, particularly in the late post-hospitalization period. Because affect balance decreases maladaptive emotion-focused coping, it indirectly weakens negative affect. At the same time, it should be noted that experiencing both kinds of emotions in a stressful event is necessary and that their dynamics and proportional changes (affect balance) over time are a testament to adaptation.
